# ON/OFF Switchable Nanocomposite Membranes for Separations

**DOI:** 10.3390/polym12102415

**Published:** 2020-10-20

**Authors:** Taegyun Kwon, Jinyoung Chun

**Affiliations:** 1 Department of Chemical and Biomolecular Engineering, Korea Advanced Institute of Science and Technology (KAIST), Daejeon 305-701, Korea; stupetegens@gmail.com; 2 Energy & Environment Division, Korea Institute of Ceramic Engineering & Technology (KICET), Gyeongnam 52851, Korea

**Keywords:** nanocomposite, membrane, reversibility, permeability, selectivity, stimuli-responsive

## Abstract

Although water, air, and other resources are abundant on earth, they have been subjected to strict environmental regulations. This is because of their limitation of availability for human consumption. In the separation industry, the membrane system was introduced to increase the amount of resources available to mankind. Experts used an easy-to-use polymeric material to design several membranes with porous structures for wastewater treatment, gas separation, and chemical removal; consequently, they succeeded in obtaining positive results. However, past polymeric membranes exhibited a chronic drawback such that it was difficult to simultaneously augment the permeate flux and improve its selectivity toward certain substances. Because of the trade-off relationship that existed between permeability and selectivity, the membrane efficiency was not very good; consequently, the cost-effectiveness was significantly hindered because there was no other alternative than to replace the membrane in order to maintain its initial characteristics steadily. This review begins with the introduction of a polymer nanocomposite (PNC) membrane that has been designed to solve the chronic problem of polymeric membranes; subsequently, the stimuli-responsive PNC membrane is elucidated, which has established itself as a popular topic among researchers in the separation industry for several decades. Furthermore, we have listed the different types and examples of stimuli-responsive PNC membranes, which can be switched by external stimuli, while discussing the future direction of the membrane separation industry.

## 1. Introduction

Most industries struggling to achieve high economic efficiency often ask researchers the following two questions:

Can two different properties be included in one material? 

If possible, can the substance be made renewable?

In other words, it is a priority to develop new materials with both multifunctionality and recyclability (reversibility) at a low unit price. However, it is virtually impossible to make such an ideal material. If a material has two opposite properties, it is generally likely that the properties exist as a trade-off of one another [1,2,3,4,5]. Because molecular-level properties can affect each other, the co-existence of two conflicting characteristics in a single material is never natural, and most materials have not done so throughout Earth’s long history. In addition, even if a recyclable material is developed, there is a limit to the number of times it can be reused [6]. Perfect permanence is difficult to achieve. As the number of reuses increases, the initial characteristics that were designed as advantages become weaker and eventually disappear. Furthermore, the requirement that materials should be inexpensive despite their novelty makes researchers raise white flags instead of experimental tools.

Sensible researchers have come up with realistic alternatives instead of rejecting unrealistic industrial requirements. Their plan is to develop relatively inexpensive new materials [7,8,9,10], but to use a mixture of preexisting materials and design products such that they can perform only under certain conditions where external stimuli are transmitted so that frequent recycling processes are unnecessary. As such, two materials with conflicting characteristics—one organic with a stimuli-responsive switching property and the other inorganic—have been combined to create a “smart” response nanocomposite system [11,12,13].

**Figure 1:**
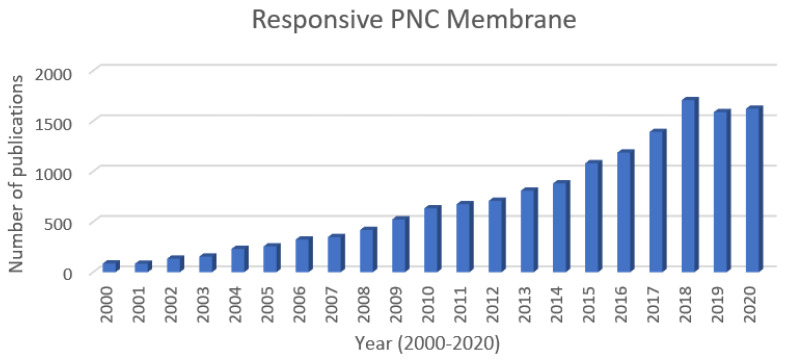
Growth of scientific output from 2000 to 2020 available at National Digital Science Library (http://www.ndsl.kr/).

Figure 1 shows the number of papers published annually in the last 20 years under the theme of “responsive polymer nanocomposite membrane”. Approximately 15,000 papers have been searched, and the number of papers has been steadily increasing every year. These publications include the results of various studies on polymer nanocomposite membrane that reacts with various kinds of external stimuli—such as light, thermal, and pH—and their applications are innumerable, such as waste water treatment, gas separation, chemical removal, and drug delivery. All these studies were obtained by the switchable molecular segments interacting with polymer chains, exposing the inner-hidden nanoparticles to the surface or isolating them from the outside.

In this review, we address the manufacturing process of nanoscale-controlled polymer nanocomposite (PNC) materials, and their application as membrane in the separation industry. Furthermore, the current level of stimuli-responsive membranes that reveal their unique characteristics only under certain conditions is reviewed.

## 2. Background 

### 2.1. Nanocomposite Systems

Organic and inorganic materials have long been regarded as conflicting materials: many believed that their domains were separate and would never overlap. In fact, at one time, scientists put forward incompatibilities between the two types of materials as evidence to support their claims. These two types of materials, whose surface properties were so different, could never be located in a single space without phase separation, and even if they could be mixed, they co-existed for only a short period of time as a mixture, not a true complex.

Many scientists began to break down minerals into smaller pieces to increase the duration of coexistence of the two materials [14,15,16,17]. The goal was to minimize the separation of the phases by overcoming gravity, and the only way to accomplish this was to expand the surface area per unit volume to increase the area at the border of the two materials. They wanted to disperse the shredded guest minerals inside the organic matter as a host matrix for as long as possible. However, this was not enough to make the mixture of the two materials a composite. For organic and inorganic materials (or dissimilar materials) to coexist perfectly, another change in the system is needed in addition to the surface area increase of the inorganic materials.

Researchers who were constantly reducing the size of their minerals to obtain more complete composite materials eventually reached the door of the nanoworld. In order to squeeze through its narrow doors, they had to break their existing habits. They decided to break away from the stereotypical ‘top-down’ approach and try something new. This was the birth of the ‘bottom-up’ method. Apart from the physical and mechanical method of breaking and grinding large materials, only through a chemical bottom-up approach, in which precursors are reduced, creates nanoscale inorganic particles that could be well-dispersed [18,19].

The particles varied greatly in size and shape, or surface conditions, depending on the composite conditions (e.g., temperature, time, type, and content of the capping agent). In addition, the particles synthesized by the sol–gel method significantly improved the dispersion stability over time compared to past methods, when the top-down approach was preferred. This is due to the presence of traces of precursors or a significant amount of organic groups originating from the capping agents on the surface of nanoparticles (NPs) at the molecular level. The nanocomposite system, which freely crosses the boundary between organic and the inorganic, has become a multi-faceted object of interest and a prerequisite for the development of hybrid materials.

Researchers who had been looking forward to the emergence of a real nanocomplex system immediately set their next goal. This was the task of fitting the organic groups present on the surface of inorganic NPs to the surrounding environment as much as possible via surface modification. They changed the molecular structure of pristine organic groups on the surface into substances that were friendly to the surrounding organic environment while controlling the surface coverage (density). The organic shell is designed tightly that the bare surface of the NP core is not visible, while sometimes it is designed sparsely that the bare surface of the NP core is visible; therefore, they can be used for various purposes in industry by finely tuning the surface coverage of NPs [20,21,22].

**Figure 2:**
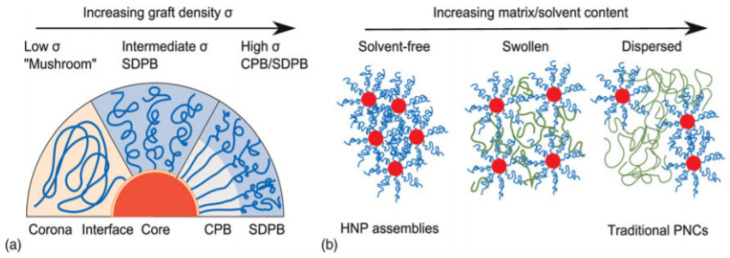
(**a**) Structure of hairy nanoparticles (HNPs) at different graft densities. At grafting densities, σ, of less than Rg−2, the corona is in a loosely coiled ‘mushroom’ conformation. As the grafting density increases, the chains are forced to extend first into a semidilute polymer brush (SDPB), and then into a concentrated polymer brush (CPB) conformation. In contrast to flat surfaces, particle curvature implies that the area per chain increases with distance from the surface, and thus the outer ends of the chain are less crowded and may transition to the SDPB regime if long enough. (**b**) The mesoscopic continuum from pure HNP assemblies (left) where the only swelling of the corona is by the corona of neighboring particles, to traditional polymer nanocomposites (PNCs) (right) where the particles are dispersed or suspended in an organic matrix. These mesoscopic structures form the basis for macroscopic morphologies whose hierarchy of structure depends on their spatial arrangement, shape, and relative volume fraction of HNP and free matrix (reprinted with permission from [23], Copyright 2013, Cambridge University Press).

NPs in core/shell structures were considered pure organic matter as the density of the organic shell surrounding NPs increased, while at the same time their affinity with the surrounding organic matrix gradually increased, which came as a shock to many researchers. Afterwards, scientists were able to develop an outstanding nanocomposite system with finely tunable miscibility through a simple mixing process that did not require any additional processing. By tailoring the nanocomposite system through NP surface modifications for various applications, they gradually solved the chronic issues they had encountered when the inorganic and organic materials were only roughly mixed (Figure 2) [23,24,25,26,27,28,29,30].

### 2.2. Preparation of Nanocomposites 

#### 2.2.1. Surface Grafting (Grafting to/Grafting from)

**Figure 3:**
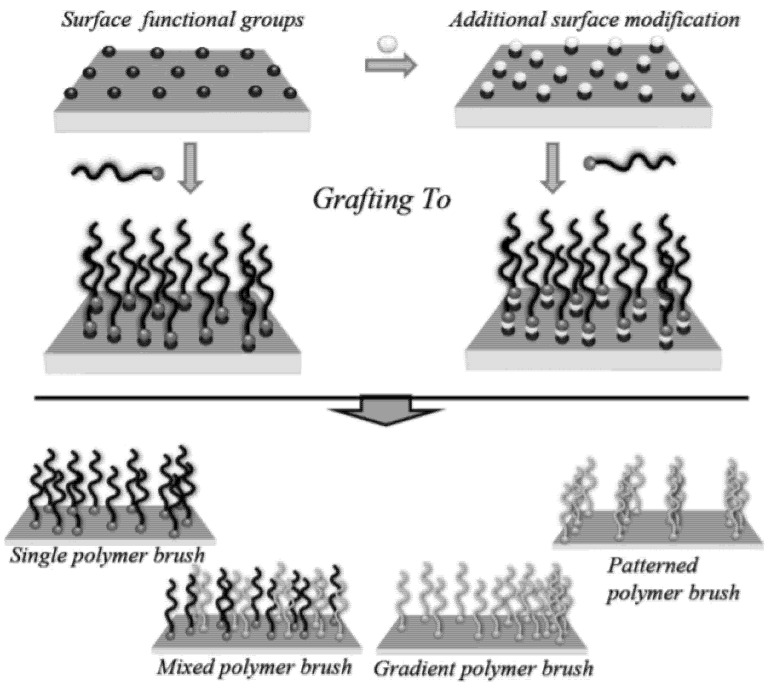
Schematic representation of the grafting-to method (reprinted with permission from [32], Copyright 2011, John Wiley & Sons).

There are several ways to implement a nanocomposite system where inorganic parts as guests and organic parts as hosts coexist, but the easiest way is the “grafting-to” method through interaction (e.g., chemical bonding, physical attachment) between the specific functional group in the preexisting organic material and the active surface site of NPs (Figure 3) [20,22,31,32,33,34,35,36]. Considering the processability, it would be beneficial to be able to attach NPs to desired locations within organic molecules via a simple mixing process. Response times, which take a few minutes at the very least and a few days at the most, have the potential to attract industry attention.

However, this method is only valid for systems that do not necessarily have to increase the density of the organic shell covering the inorganic NP surface. This is because the large steric hindrance between organic chains makes it virtually difficult to attach large amounts of organic brushes to the surface of NPs. Although there are a considerable number of active sites (i.e., functional groups) within organic molecules, these functional groups are simply useless if each of the organic molecules occupies a large volume.

The disadvantages of the grafting-to method are low attachment capability and low grafting density on NP surfaces, and several studies have been conducted globally to improve the affinity with the organic matrix by increasing the grafting density.

**Figure 4:**
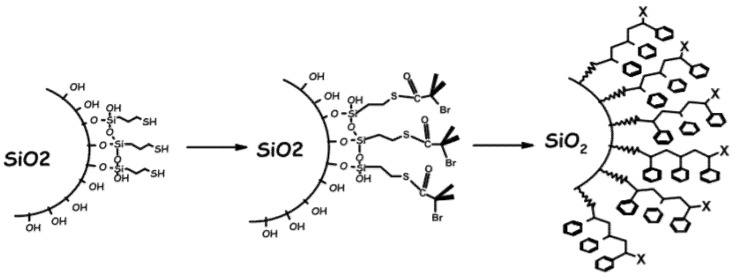
Grafting of the initiator molecule via the ‘over-grafting method’ (reprinted with permission from [39], Copyright 2004, Elsevier).

Researchers have planted and grown seeds (i.e., small molecules for leading polymerization) instead of planting bulky trees (i.e., as-prepared polymer brushes) as a way to increase the grafting density. The grafting-from method involves attaching small molecules, which have almost no steric hindrance, to the surface of NPs first and attaching the monomers one by one to form a polymer. Polymer chains, which grow from the surface of NPs through radical polymerization (i.e., reversible addition-fragmentation chain transfer), showed higher grafting density compared to the grafting-to method (Figure 4) [37,38,39,40,41,42,43,44,45,46]. Moreover, this method did not require the final washing step to remove the unattached free polymer, which was pointed out as a tricky finishing process in the grafting-to method, thus relieving researchers of the burden. The monomer molecules with very small molecular weights were easily dissolved in the reaction solvents, so no cumbersome purification steps were necessary.

The ‘grafting-from’ method goes a step further from forming a dense organic shell around NPs and fabricates the organic matrix itself with NPs. This fabrication method for nanocomposite material also called ‘in-situ interfacial polymerization’ from the NP surface.

#### 2.2.2. In-Situ Interfacial Polymerization

This method is not much different from the grafting-from method in terms of polymer growth from the surface of NPs [47]. However, unlike the grafting-from method, in which each NP with a core/shell structure is used as a nanocomposite material, the in-situ interfacial polymerization method is applied from the outset to form a polymeric thin film with NPs, so the application is slightly different. In addition, the grafting-from method begins with attaching a chain transfer agent (CTA) to the surface of NPs, while in-situ interfacial polymerization starts with organic agents such as silane coupling agents attached to the surface of NPs. For example, many researchers reported that NPs—such as TiO_2_, SiO_2_, and zeolite—were used as guests for mixing with in-situ polymerized matrices; these complex materials are often used to manufacture forward osmosis (FO) membranes. This means that in-situ interfacial polymerization can be applied in various fields as a manufacturing method for nanocomposite membranes [48,49,50,51,52,53]. 

Recently, many studies have been conducted to improve the weaker interaction between NPs and the polymer matrix through in-situ interfacial polymerization based on the grafting-from technique, and the developed nanocomposite materials are receiving considerable attention for their ability to improve the hydrophilicity and anti-fouling effects. Surprisingly, these nanocomposites are free from trade-off properties, cited above as the chronic weakness of the traditional membrane materials [54,55,56,57].

**Figure 5:**
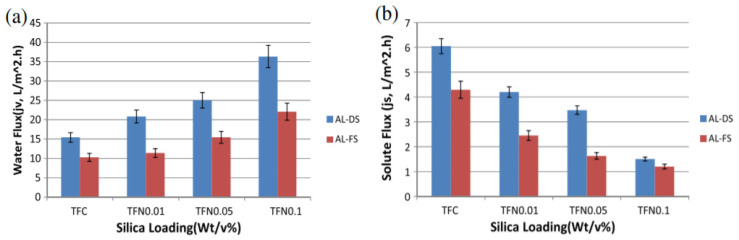
FO water flux and solute flux of synthesized FO membranes. (**a**) FO water flux and (**b**) solute flux (reprinted with permission from [50], Copyright 2014, Elsevier).

Amini et al. bonded monomolecules to photocatalytic TiO_2_ NPs with a surface modified with 3-aminopropylithylane (APTES) and combined them with polysulfone substrates [48]. In the same way, Niksefat et al. used 1,3-phenylenediamine as a medium for connecting SiO_2_ NPs with as-prepared polysulfone substrates to manufacture polymeric membranes with thermo-stable templates (Figure 5) [50]. In addition, Ma et al. first introduced NaY zeolite NPs, which have never been reported before, as guests of the nanocomposite membrane system, and succeeded in manufacturing thin film nanocomposite (TFN) through in-situ interfacial polymerization. Their study has great significance in that it has informed many researchers that there are still many unknown combinations in the membrane industry, as well as the fact that the zeolite-incorporated membrane showed a characteristic that surpasses the existing membrane performance [51]. 

Organic polymers and inorganic nanoparticles, which had been difficult to stay together in the same space, were able to take a step closer to each other showing a uniform homogeneous phase despite each of the completely different surface characteristics. The PNC system, which was born through these processes, was able to be free from phase separation having both polymer’s hydrophobic properties and nanoparticle’s hydrophilic properties. The PNC system, which has become an ideal material that everyone has so desired, knocked on the door of the environmental sector (especially the separation industry) which is eager to efficiently purify the polluted air and waste water [58,59,60,61,62,63,64]. Before long, it was able to become unique as one of the outstanding membrane materials.

## 3. Applications for Membrane Systems 

### 3.1. Membrane Fabrication with Surface-Modified NPs

A nanocomposite system consisting of NPs with hydrophilic surfaces and hydrophobic organic materials is very useful in many areas, especially in the field of separation processes (industry, medicine, environmental sciences, etc.) for water treatment. Nanocomposites are attracting attention as alternative materials for various kinds of membranes (e.g., ultrafiltration, nanofiltration, microfiltration, forward osmosis, and reverse osmosis) by owing to the characteristics that they can provide a significant number of hydrophilic sites within the matrix through the active surface of NP guests.

It is true that conventional membranes without NP guests have been evaluated as specialized filters that can purify large amounts of material while only allowing certain materials to pass through by changing the fluid according to the size of the membrane pores. However, the fact that these specialized filters have some fatal problems has also become well known. Above all, most ingredients for the preparation of porous membranes—including polyethersulfone (PES), polyvinylidene fluoride (PVDF), and polypropylene (PP)—consist of hydrophobic segments. Membranes composed of these substances are vulnerable to adsorption of hydrophobic pollutants (e.g., proteins, humic materials, and microorganisms) [65,66,67,68].

**Figure 6:**
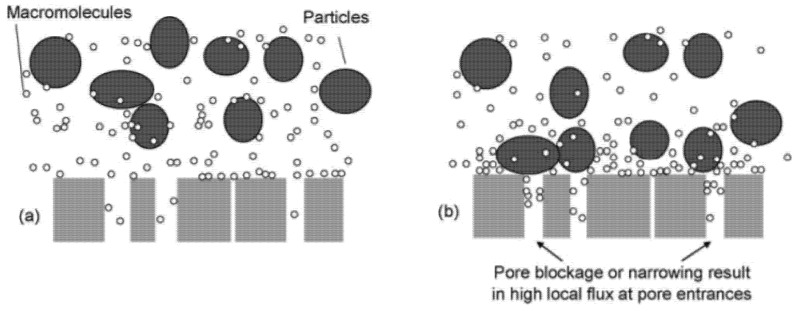
Progressive pore blockage leading to rapid TMP increase. (**a**) The initial low fouling phase and (**b**) enhanced rejection of macromolecules and deposition of larger particles by the pore closure (reprinted with permission from [66], Copyright 2006, Elsevier).

Pollutants adsorbed on the surface of the membrane by hydrophobic interactions cover the pores of the membrane (i.e., membrane fouling), eventually forming a film and sharply lowering the permeability efficiency of the membrane for a short period of time (Figure 6). The initial pore size becomes smaller over time, and the materials that can pass through the smaller pores become more limited [69,70]. To regain the initial efficiency of the membrane, the process of constantly rinsing out unwanted pollutant was necessary. This process was very tricky and uneconomical. Those who longed for the development of the separation industry continued to search for ways to minimize the need for cleaning membranes [71]. 

Many researchers have proposed ways to prevent the membrane fouling effect by introducing hydrophilic sites by incorporating NPs into membrane materials [51,66,72,73,74,75,76,77,78,79]. For example, the aforementioned research by Ma et al. inhibited the fouling phenomenon by incorporating hydrophilic zeolite NPs into hydrophobic polysulfone. The zeolite used in this study was evaluated by many as an effective system because it had a porous structure and could contain numerous hydrophilic sites with large surface areas [80]. Mesoporous carbon materials, such as carbon nanotube, were also able to enhance the hydrophilicity of the membrane by mixing with hydrophobic matrix as a bulk supplier of hydrophilic sites [81,82]. As such, the PNC membrane was able to successfully prevent the adsorption of pollutants owing to NP guests with a hydrophilic surface, and it was established as one of the outstanding candidates with anti-fouling performance in the contemporary membrane industry. 

**Figure 7:**
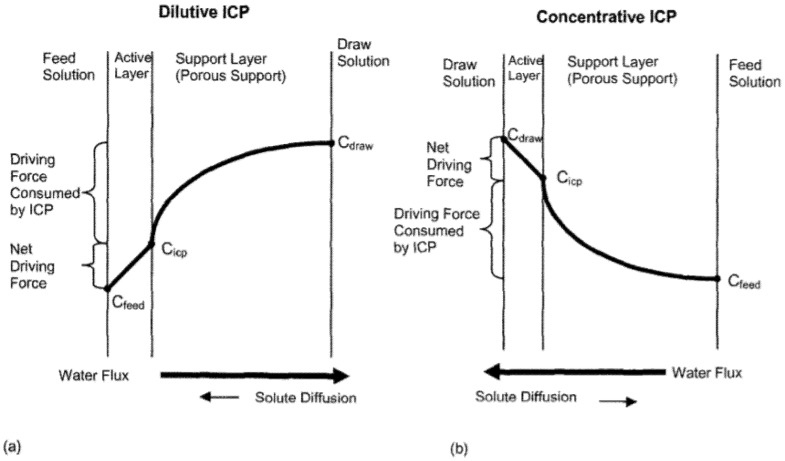
Schematic representation of (**a**) dilutive internal concentration polarization (ICP) and (**b**) concentrative ICP (reprinted with permission from [83], Copyright 2006, Elsevier).

Meanwhile, the PNC membrane has been spotlighted as a material that can alleviate internal concentration polarization (ICP), which, along with the membrane fouling effect, is considered another drawback of traditional membranes. The ICP phenomenon arises from competition between the two main properties of the membrane—namely, permeability and selectivity. Substances that fail to pass through the pore accumulate at the surface of the membrane, obstructing the movement of the permeate at some point and worsening the degree of stacking over time. It is known that the generated concentration gradient causes ICP, and when the accumulation is not alleviated and a certain period of time elapses, the mixture of residue and permeate causes the aforementioned fouling phenomenon (Figure 7) [83,84,85].

Many researchers have stated that the structural parameters (S) in the porous support layer should be reduced to minimize this ICP phenomenon during the FO process [86]. Three methods have been chosen to lower the structural parameters (S) defined by the [thickness × tortuosity/porosity] of the porous layer: a reduction in thickness, decrease in tortuosity, and increase in porosity. In one early study, the material lowering the S value was a nanofiber consisting of organic materials. However, nanofibers have the disadvantage of having very low mechanical strength, and the problem was that it took a very long time for a substance to pass through.

The alternative to nanofibers was none other than the membrane system, in which certain inorganic NPs were distributed evenly. In order to increase the porosity of the support layer, porous zeolite NPs were chosen as guest materials for the system, thereby creating an ideal membrane layer that meets the purpose of increasing the mechanical strength and water flux and minimizing the ICP phenomenon [80,87].

Recently, many researchers have discovered new microfabrication (UF) membrane materials that go beyond the combination of zeolite NPs and polymeric films and have reported surprising results [88,89,90,91,92,93]. For example, a study that incorporated SiO_2_ NPs within poly(4-methyl-2-pentyne) (PMP) to maintain the permeability of n-butane while simultaneously increasing the selectivity of n-butane/methane suggests that PNC plays a major role in solving the existing problems of membranes [26,94,95,96,97,98,99,100]. PNC membranes have been in the spotlight in the separation technology field in recent years as ideal membrane materials that can interfere with the hydrophobic interaction between the porous membrane and the pollutant. This interest grew further when the PNC membrane began to feature the conative ON/OFF switching system. 

### 3.2. Reversible Smart Membranes

Researchers have evolved separation technology in terms of PNC membrane activeness and reversibility. A system in which externally transmitted stimuli act as triggers, and the specific molecular structure contained in the polymer matrix acts as a switch called a “stimuli-responsive reversible smart membrane”. When stimuli are delivered from the outside, the membrane exposes the NP guest to its surface as if it were alive, and once the stimuli disappear, the NP guest hides again. In other words, it is a smart membrane system, as the name implies [101,102].

Many researchers who hoped to activate the hydrophilic potential of the membrane only when they desired incorporated an ON/OFF switch inside the membrane that responded to only a few specific stimuli (e.g., light, heat, chemical, electronic fields, mechanical fields, and mechanical force). As the polymer chains swell, the hydrophilic surface of NP is hidden and isolated from the surrounding environment, and, on the contrary, when the polymer chains collapse, the hydrophilic surface of NP gradually manifests, demonstrating its capacity as a PNC membrane. Because the system is sensitive to changes in the surrounding environment, it is finely self-controlled without having to worry about it, and in some cases, it does not lose its initial reversible efficiency even after a long period of operation [103,104,105].

Many researchers have designed several switchable PNC membrane systems by introducing molecular structures suitable for given circumstances and experimental purposes. Some typical systems with responsive ON/OFF switches are grouped according to external stimuli.

#### 3.2.1. Photo-Responsive Membranes

Photo-responsive systems are largely divided into two types, depending on what the subject of photoexcitation is. If the NP guest acts as a subject that facilitates chemical reactions in response to light having a specific wavelength, it is classified as photocatalysis, whereas if the photochromic segment contained in the polymer matrix is the main body that reacts to light, it is called photoisomerization. This review does not address the photoexcitation of the NP material itself; it only addresses the reactivity of the polymer matrix surrounding the NPs. Moreover, because most of the reported photo-responsive materials in the reversible smart membrane field are driven by photoisomerization phenomena of the polymer matrix, a description of NP photoexcitation (photocatalysis) will be replaced by relevant references [106,107,108,109].

Many researchers hope that the range of light wavelengths being stimulated would not deviate significantly from the visible light range when it comes to selecting materials for photoisomerization. The farther away the sensitive range is from visible light, the less applicable it will be, and no matter how fast the response speed and how high the isomer conversion rate, it is shunned by the industry. 

Two representative materials that successfully passed the demanding verification work are azobenzene and its derivatives. The azobenzene segment, which exists in two forms of isomer depending on the wavelength of light, is considered the best photochromic moiety for photo-responsive membranes. In addition to reacting to light (e.g., UV, visible, and NIR) of easy-to-apply wavelengths, the conversion rate between cis-/trans-, which is not only quick-response but also near-perfect, was enough to attract the attention of industry workers.

Researchers who observed, with their own eyes, changes in the molecular structure of azobenzene converted from trans- to cis-form or cis- to trans-form under the irradiation of UV and visible (or NIR) light, respectively, designed a photo-responsive PNC system with a core/shell structure by placing an azobenzene segment around NPs. The system was able to control its own dispersibility by switching back and forth between cis- and trans-form, depending on the wavelength of light being irradiated [110,111]. For example, Wei et al. prepared a photo-sensitive shell by introducing a ligand containing an azobenzene moiety on the surface of AuNPs to implement the reversible catalysis effect. Subsequent investigations using UV and visible light resulted in successive aggregation and dispersion of NPs, and these changes in dispersibility resulted in the deactivation/activation of the catalytic site, respectively.

**Figure 8:**
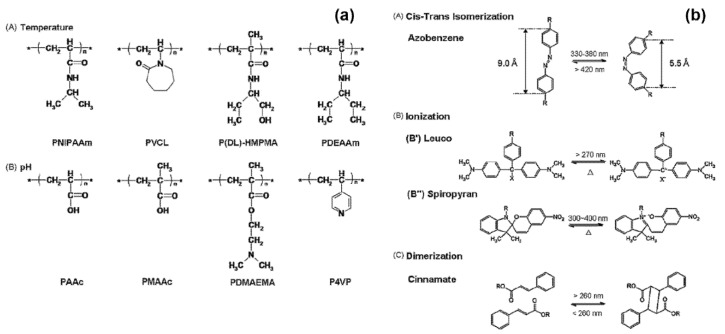
(**a**) Examples of molecular structures responsive to temperature (A) and pH (B). (**b**) Examples of molecular structures of photo-responsive monomers: cis–trans isomer of azobenzene (A); ionization monomers (B) of leucos (B’) and spiropyran (B”); and dimerization monomer of cinnamate (C). (III) Orientation changes (A) and molecular structures (B) of liquid crystalline molecules (reprinted with permission from [124], Copyright 2010, Elsevier).

On the other hand, many researchers have applied azobenzene in a different way, in which the shape of the molecule changes uniquely depending on the wavelength of light, such as the size change of tubular pores in the membrane. These researchers expected to be able to selectively filter out the substances passing through as the membrane’s pore size changed, and their wishes soon became reality. The PNC membrane system with the introduction of azobenzene molecules has since been widely used as a photo-sensitive drug-releasing model [112,113,114,115]. For example, Lu et al. combined azobenzene derivative molecules on the pore surface of a mesostructured silica nanoparticle so that dyes and drugs contained within the system could escape only under certain wavelength conditions. Yan et al. also utilized the PNC membrane system as a photo-sensitive nanocarrier by attaching azobenzene-based rotaxane to the surface of Au nanorods and including drugs. Several representative photo-sensitive moieties—including azobenzene, and their working mechanism—are shown in Figure 8.

#### 3.2.2. Thermo-Responsive Membrane

Thermo-responsive systems are largely divided into two categories, direct and indirect, depending on the mechanism of receiving heat. This category encompasses systems that are directly affected by temperature changes in the ambient environment and systems that indirectly utilize heat generated from other external stimuli (e.g., light, magnetic field). However, this classification is often ambiguous because it is simply based on the different ways heat is supplied.

Many researchers suggest the pattern of phase transition (volume variation) with temperature changes as another criterion for classifying systems. When the ambient temperature rises, most polymers tend to exhibit so-called upper critical solution temperature (UCST) behavior, in which the polymer increases in volume above a certain temperature. This is because polymer chains swell at higher temperatures, which is an intuitive phenomenon.

On the other hand, some polymers do not follow this natural phase transition process, but rather the reverse. As the temperature increases, the polymer chains shrink, reducing the overall volume. In other words, these polymers exhibit lower critical solution temperature (LCST) behavior. This is because there is a specific moiety inside the polymer chain that can be bound via hydrogen bonding. As the temperature increases and the attractive force with the solvent molecules (i.e., hydrogen bonds) decreases, the polymer chains push the solvent molecules and shrink, resulting in a decrease in volume. Such hydrogen-bonded polymers that exhibit LCST behavior can fully reveal their reversibility with temperature changes within aqueous solution. These polymers are widely used as materials for water purification or in drug-transfer membranes. Moreover, the fact that the phase transition point of the UCST polymer is higher than that of the LCST polymer is another reason why most researchers focusing on thermo-responsive membranes have a preference for certain LCST polymers.

At temperatures above the transition point, NPs were covered with swollen polymer chains, concealing the original characteristics of the NPs, while the polymer chains attached to the NP surface shrunk when the surroundings were cooled below the transition point and the NPs were repeatedly exposed to the outside again, making the process perfectly thermo-reversible. For example, Xiao et al. reported a significant difference in the water flux passing through the membrane at temperatures higher and lower than the transition temperature by combining a poly(N-isopropylacrylamide) (PNIPAAm) polymer with PVDF, which shows LCST behavior and has a transition temperature of 32 °C [116]. This result was expected to some extent based on the Hagen–Poiseuille law, in which the amount of permeate flux varies depending on the pore size [117]. On the other hand, many researchers have directly changed the size of the pores in the existing porous membrane template using the reversible volumetric changing ability of the PNIPAAm molecule, and as a result, they have succeeded in selectively filtering the permeates.

In addition, some researchers have introduced additional moieties (hydrophilic or hydrophobic) to the PNIPAAm molecule to change the working range by changing the transition point of the existing 32 °C [118] and attaching certain segments to control the working speed [119]. For example, Xie et al. introduced an acrylamide monomer as a hydrophilic moiety to increase the LCST of PNIPAAm to 40 °C, while introducing butyl methacrylate monomers as hydrophobic moieties to lower the existing LCST to 17.5 °C [119]. In addition, Kaneco et al. grafted the PEG segment to the PNIPAAm molecule so that the volume change of PNIPAAm occurs faster than usual [120]. 

Many polymers besides PNIPAAm are frequently used as materials for thermo-responsive membranes, and they are also performing a role as materials for smart membrane systems by changing their volumes at their respective transition temperatures. Some representative thermo-responsive moieties are depicted in Figure 8. 

In addition, there are many studies regarding the sol–gel phase transition, another known mechanism in thermo-responsive systems. However, since it does not differ much from the volume change process described earlier, we simply provide references to existing literature [121,122,123]. 

#### 3.2.3. pH-Responsive Membranes

The membrane system, in which the pH of the aqueous solution acts as a trigger for the beginning of the reaction, is accomplished through the process of obtaining or losing the proton of the ionizable functional groups contained inside the organic material. Some polymers increase in volume due to electrostatic repulsion between ionized functional groups [124,125]. At this time, external substances can pass through the gap between the polymer chains, which many researchers have used to modify the permeability characteristics of the membrane. For example, Zhan et al. reported that when changes occur in the ionization state of the polydopamine (PDA) surrounding the Pt-MnSi NP, the active surface site of the NP core is exposed to the outside, resulting in catalysis [126]. 

In addition, a structural change occurs within the polymers, which swell or shrink according to the surrounding pH value. This change has a great effect on the hydrophilicity of the membrane. In other words, it is possible to change the surface energy of membrane. This means that the anti-fouling effect, previously referred to as the basic characteristic of PNC membrane, can also be granted additional reversibility [127].

Many have maximized the reversible anti-fouling effect by increasing the difference in surface energy between the swelled state and the collapsed state of the pH-responsive polymer chain [74,128,129,130]. For example, Hernandez et al. introduced ionizable polyacrylic acid (PAA) as a pH-sensitive organic part, and selected PVDF with very low surface energy as a membrane template for grafting [130]. Some commonly used pH-responsive materials are shown in Figure 8.

### 3.3. Limitations and Expectations

As already shown in Figure 1, the market for the stimuli-responsive PNC membrane has grown steadily over the last 20 years, and the findings involved are surprising the world with the release of a series of studies, but unfortunately, the rate at which industry requirements are diversified is always faster than the pace at which researchers develop. It has already been decades since the membrane systems, which include the coexistence of hydrophobic/hydrophilic properties as well as the molecular ON/OFF switch, were developed and the industry’s hot response has cooled off. The requirements of those engaged in the separation industry are largely divided into economics and diversity.

Those seeking economic feasibility feel sorry for the materials so far, especially the intrinsic high cost of inorganic nanoparticles. They have experienced the outstanding effects of TiO_2_ NPs [131,132,133,134], Ag NPs [135,136,137], and GO [138,139,140], which had given PNC membranes hydrophilic properties, but they are eager to develop more economical materials at a time when mass production is imminent. Of course, small-scale studies of lab scale introduced by less expensive nanoparticles—such as ZnO [141,142], CaCO_3_ [143,144], zeolite [80,145], and HNTs [57,146]—are being conducted in this regard. Without steady excavation of additional materials following these studies, growth in the membrane market, which has been shown so far by the stimuli-responsive PNC material, is bound to be limited.

In addition, those seeking diversity have complained that there is only one kind of stimulation for switching triggers. Unfortunately, however, the types of molecules that can accept the stimulus of the environmental are extremely limited, and most of the molecules respond only to one stimulus. Of course, if we list all the molecules on Earth that have not yet been reported to academia, we may add a few more kinds. Some researchers who were not able to wait for new sensitive molecules that might emerge, and others who were tired of the existing smart system responding to only one stimulus, developed a ‘smarter’ system named multi-stimuli responsive system by simultaneously including segments that respond to two or more different stimuli in one system [147,148,149]. For the same reason, the previously introduced several researchers changed the environmental reactive range by slightly touching the structure of the stimuli-sensitive molecule [119]. However, this is also not as active as expected because of the limited number of applicable molecules. In order to keep pace with the ever-increasing demands of the industry, research must be actively conducted to develop new sensitive segments and modify existing responsive molecules; in addition, the newly born sensitive molecules must have rapid response speed and recyclability close to perfection.

## 4. Summary

Filtration technology using membranes has been studied in the direction of increasing permeate flux as well as providing permeate selectivity to the pore used as the moving channel of the substances. However, these two characteristics gradually diminished as the driving time of the membrane elapsed. This is due to substances that have not yet passed through and remain near the pore, being accumulated or adsorbed. Faced with these problems, researchers have devised a stimuli-responsive PNC membrane system to improve the permeability and selectivity of the membrane while extending the retention period of the early state. They increased the filtration efficiency of the membrane by combining hydrophilic inorganic nanoparticles with a hydrophobic polymer matrix and by introducing a stimuli-sensitive moiety as a molecular ON/OFF switch on the surface of the pore. Currently, many researchers are trying to introduce and combine various materials to further improve the response rate and purification efficiency of existing stimuli-responsive PNC membrane, and have so far continuously reported their findings.
